# Unleashing the green potential: Unraveling the power of environmental concerns in driving employees’ green behavior

**DOI:** 10.1371/journal.pone.0320053

**Published:** 2025-03-25

**Authors:** Nhon Hoang Thanh, Bac Truong Cong

**Affiliations:** 1 Faculty of Commerce, Van Lang University, Ho Chi Minh City, Vietnam; 2 University of Economics and Law, Ho Chi Minh City, Vietnam; 3 Vietnam National University, Ho Chi Minh City, Vietnam; Southwestern University of Finance and Economics, China

## Abstract

This study investigates how environmental concerns influence employees’ green behavior within businesses, with a focus on the roles of attitude, green knowledge, and perceived behavioral control, underpinned by the Theory of Planned Behavior (TPB). Guided by TPB, this study develops a model where environmental concern impacts employee green behavior both directly and indirectly through its influence on attitudes, perceived behavioral control, and green knowledge. The study employed a structured survey of companies listed on the Vietnam Stock Exchange, targeting directors and senior managers between April and December 2021. A total of 407 valid responses were analyzed using structural equation modeling. The findings reveal that environmental concerns, attitudes, and perceived behavior control significantly influence employees’ green behavior. Both attitudes and perceived behavior control act as positive mediators in the relationship between environmental concern and employees’ green behavior. Additionally, the study highlights that the level of environmental consciousness is a crucial factor in shaping the connections between attitudes, perceived behavior control, and employee green behavior. These insights offer a novel perspective on the complex dynamics of environmental concerns in the corporate workplace, paving the way for the development of strategies that promote environmentally friendly behaviors within organizations.

## Introduction

Social concerns relating to the greenhouse effect, a major driver of climate change, are steadily increasing [1]. Although there is an urgent need for the business community to act quickly to prevent the potentially severe impacts of climate change on environmental sustainability, it is not easy to put pressure on both employer and employee to protect the environment if such environmental protection activities do not bring benefits for them [2]. Realizing the important role of the workforce in maintaining environmental sustainability, environmental protection entities and policymakers have been organizing various conversations around the world to discover and define what it means to encourage a green workforce. This is because carbon emissions and waste disposals caused by employee activities, such as driving to the workplace and using air-conditioners and disposable materials, contribute to the detriment of the environment [3]. Therefore, it is important to train employees to perform green behavior to help the company operate in environmentally sustainable ways.

According to Sabbir and Taufique, employee green behavior is defined as “scalable actions that employee engages in that contribute to environmental sustainability” (p.112) [4]. In addition, employee green behaviors are intentional behaviors that play an important role in minimizing the negative impacts of activities of employees at work on the environment [5]. It is confirmed that employee green behavior has positive effects on environmental sustainability [6–8]. However, efforts to enhance environmental performance face challenges due to individuals working for the same company differing in terms of environmentally responsible behaviors. Psychological elements, including attitude, green knowledge, and perceived behavior control mentioned in the Theory of Planned Behavior (TPB), play a crucial role in elucidating the reason why there are different levels of employee involvement with green behavior [9]. Attitude in this study refers to “the degree to which a person has a favorable or unfavorable evaluation of the behavior of interest.” (p. 26) [10]. Green knowledge pertains to the understanding possessed by employees about environmental protection [3]. Perceived behavior control refers to “a person’s perception of the ease or difficulty of performing the behavior of interest.” (p. 25) [10]. Prior studies confirmed that the positive influence of psychological factors on green behavior may help companies reinforce the environmentally conscious actions taken by employees in manners that exemplify their environmental concerns [3,11]. Previous research also indicated that employee’s environmental awareness has increased dramatically over the past decade [6]. Environmental concern refers to one’s perception, knowledge, and belief about environmental issues, to what extent a person is concerned for and is worried about threats to the environment, and supports efforts to solve them [3]. Therefore, studies contend that environmental concern, along with attitude, green knowledge, and perceived behavior control, has a significant impact on employee green behavior [3,11,12].

While research on environmental consciousness has grown, most studies have concentrated on consumer green behavior or sector-specific environmental practices, such as those in hospitality [13, 14] or education sectors [15–18]. There remains a notable gap in the literature on how managerial perspectives influence green behavior within corporate environments, particularly in emerging economies like Vietnam, which is one of the largest population countries in Southeast Asia and enjoys unique cultural and socio-economic characteristics. This study addresses this gap by exploring the drivers of green behavior among managers and senior employees in publicly listed Vietnamese companies on the Vietnam Stock Exchange, an under-researched yet critical group in influencing organizational green practices. Moreover, although several studies highlight the direct relationship between environmental concerns and employee green behavior, it remains unclear indirect effects and moderator roles of environmental concerns in the nexus with employee green behavior.

According to the Environmental Performance Index 2022, Vietnam has been ranked 178 out of 180 countries [19]. Environmental pollution has worsened in urban, especially in areas around industrial parks. This is because the enforcement of environmental protection regulations is still limited. Weak monitoring of the implementation of environmental protection laws is considered a big issue at the local level [19]. Hence, Vietnamese business organizations, especially manufacturers, have considered maintaining environmental sustainability as a low priority in their operations, so employees are less supported by their employers in environmental protection activities. In other words, without the actions and support of the employer toward preserving the environment, employees are not encouraged to implement green initiatives. Consequently, Vietnam suffers from low air quality, greenhouse emissions, and natural resource waste [20]. Realizing the important effects of environmental protection on sustainable developments and green growth, in 2016, the Vietnamese government devised a national action plan on environmental sustainability with a mission to 2030 to minimize the negative impacts of climate change on everyday life and aim to green economy growth.

In general, we expand upon the previous studies conducted by Fawehinmi et al. [3] to contribute to the literature by investigating how environmental concern and psychological factors, including attitudes, green knowledge, and perceived behavior control, influence employee green behavior, respectively. Likewise, we expand the study of Saari et al. [21] to enhance current insights into environmental sustainability by examining the mechanisms by which environmental consciousness indirectly influences employee green behavior via psychosocial factors. Moreover, we attempt to provide novel insights into environmental management literature by investigating the moderating role of environmental concern on the relationship between psychological factors and employee green behavior.

The remainder of the paper is organized as follows. The Literature review and hypothesis development section articulates the literature review and hypothesis developments. The Materials and methods section provides an overview of the materials and methods. In the Results section and Conclusion section, we present results, discussions, theoretical and practical contributions, limitations, and further research at the end.

## Literature review and hypothesis development

To investigate the factors influencing employee green behavior, this study adopts the Theory of Planned Behavior (TPB), developed by Ajzen [22], as the primary theoretical framework. The TPB has been widely utilized to predict intentional behavior by examining three core components: attitude, subjective norms, and perceived behavior control. Given its emphasis on intention-driven actions, TPB provides a suitable basis for understanding how employees’ beliefs shape their decisions to engage in green behaviors. In TPB, both personal beliefs and perceptions about control over actions influence individuals’ behavioral intentions, making it particularly relevant for workplace settings where employees’ actions often depend on their perception of available resources and support [23–25].

Research has shown TPB’s effectiveness in studying pro-environmental behavior in diverse contexts, including workplace settings, which supports its use in the current study [17,26]. Specifically, TPB has been successfully applied in examining employees’ willingness to engage in green practices in the hospitality industry, where environmentally friendly behaviors, such as energy conservation, recycling, and waste reduction, depend on personal attitudes and perceived control within structured organizational environments [14,27,28]. This evidence highlights the theory’s applicability for understanding green behavior in workplace settings where decision-making is intentional, context-specific, and often shaped by organizational constraints. By using TPB, this study can provide a structured and validated approach to analyze the influence of environmental concerns, attitudes, perceived behavioral control, and green knowledge on green behavior within corporate management settings.

Although other theories, such as the Norm Activation Model (NAM) or Value-Belief-Norm (VBN) theory, are popular in environmental behavior research, TPB offers unique strengths that align better with this study’s focus. NAM and VBN are often associated with moral obligations or altruistic motives, which are less applicable to structured corporate contexts where actions are more likely to be driven by planned behaviors rather than spontaneous moral imperatives [29–31]. Additionally, TPB’s emphasis on perceived behavior control aligns well with the structured and goal-oriented nature of corporate environments, where green practices often require both individual commitment and supportive resources. Therefore, TPB was selected as the most fitting framework for studying intentional and controllable green behavior among employees in publicly listed Vietnamese companies.

The study is built on the assumptions of the theory of planned behavior developed by Ajzen [22]. The theory of planned behavior, a psychological theory, is used to predict an individual’s intention to engage in a behavior at a specific time and place [32]. The theory maintains core components, namely, attitude and perceived behavior control, which shape an individual’s behavior intention [9]. Over the years, the theory of planned behavior was expanded by including other background factors, such as knowledge as a predictor of specific behavior [9]. Hence, learning green knowledge is anticipated to motivate employees to go green [33].

### Environmental concern and attitude

In TPB, attitude is a primary factor influencing behavioral intentions, shaped by prominent beliefs [22]. Prior research argued that environmental concern is promoted as a type of conviction [9,33–35]. In other words, attitude is influenced by environmental concerns [36–38]. It is posited that the level of environmental concern among employees will shape their attitude towards engaging in environmentally friendly behavior [34]. The study of Albayrak et al. espouses that individuals with deep ecological concerns would enjoy eco-friendly buying behavior [36]. Besides that, the increase in understanding the consequences of actions jeopardizing environmental sustainability would have a significant impact on attitudes toward conserving the environment [39]. Similarly, engaging in corporate activities related to environmental protection can assist employees in cultivating problem-solving skills to address environmental challenges [18]. Based on the discussions above, we propose the hypothesis as the following:

*H1: Environmental concern has a positive association with attitude.*

### Environmental concern and perceived behavior control

Perceived behavior control is supposed to be based on control beliefs [3]. A control belief is defined as “a person’s subjective probability that a given facilitating and inhibiting factor will be present in the situation of interest” [40]. Linking TPB to our research, it is argued that environmental concern is control beliefs predicting perceived behavior control [3]. Therefore, environmentally responsible or concerned employees are proactive in solving environmental problems, and they possess confidence in their green actions. In addition, the more employees are concerned about environmental issues, the more they perceive self-efficacy for conserving the environment [41]. Self-efficacy is also confirmed as a component of perceived behavior control [9], thus, perceived behavior control is affected by environmental concern [32, 33]. Besides that, Akhtar et al. accentuates that pro-environmental actions at the workplace, such as recycling paper, reducing the use of air- conditioners, and disposing of waste properly, tend to engage employees in green behavior, which ultimately enhances environmental performance [18]. Therefore, we propose the hypothesis as the following:

*H2: Environmental concern has a positive association with perceived behavior control*

### Environmental concern and green knowledge

Environmental concern serves as a significant driver of individuals’ motivation to acquire knowledge about environmental protection and sustainability [3]. Concern for environmental issues can prompt individuals to seek out additional skills and insights into ways to reduce environmental harm and promote sustainability [43,45]. This perspective defines green knowledge as an individual’s understanding of the interconnections between human actions and environmental health, encompassing a range of ecological principles and circular economy practices [42]. Research supports this link, demonstrating that individuals who hold strong environmental concerns are more likely to pursue knowledge that aids in adopting sustainable practices, such as the utilization of biofuels to reduce ecological impact [46]. Furthermore, findings show that educators in Malaysian higher education institutions contribute meaningfully to environmental preservation when they leverage their capabilities, motivation, and access to opportunities to foster both declarative and procedural environmental knowledge [44]. Similarly, studies reveal that the environmental awareness of university staff contributes to knowledge-sharing activities, advancing collective green knowledge within campus communities [47]. Thus, as employees become more concerned about environmental issues, they are likely to strengthen their knowledge base on these matters. Therefore, we propose the hypothesis as the following:

*H3: Environmental concern has a significant impact on green knowledge*

### Attitude and employee green behavior

Attitude toward specific behavior is said to be a function of behavior belief [4,48,49]. A behavior belief is an individual’s subjective probability that performing a behavior of interest will produce a positive result [4,50]. For example, the belief is that recycling paper and plastics and saving electrical energy may protect the environment. It is argued that employees’ attitude toward the environment impacts their engagement in environmental protection behaviors [49,51]. Previous studies conclude that employees with a positive environmental attitude are more motivated toward environmental preservation, ultimately leading to the display of green behaviors [52–54]. Even in cases where employees may lack extensive knowledge of environmental issues, those in manufacturing sectors still exhibit a positive attitude toward environmental conservation behaviors [55]. This highlights that attitude plays a central role in fostering and promoting green behavior [45,56], particularly within corporate settings where motivation and environmental awareness shape sustainable practices. Based on these arguments, we propose the following hypothesis:

*H4: Attitude has a significant impact on employee green behavior*

### Perceived behavior control and employee green behavior

According to the TPB, perceived behavioral control (PBC) represents an individual’s belief in their capability to carry out a behavior, considering both their confidence level and the accessibility of supportive resources. In workplace settings, PBC encompasses employees’ perception of control over performing green behaviors, such as energy conservation and proper waste disposal, which are often influenced by the availability of resources (e.g., recycling bins) and confidence in effectively carrying out these actions [39,57]. Research has consistently linked higher levels of PBC with increased engagement in environmentally sustainable practices [58]. For instance, the confidence of employees in having control over energy-saving practices correlates directly with their likelihood of reducing energy consumption [59]. Similarly, employees in higher education institutions have been found to demonstrate strong personal control in practicing eco-friendly behaviors, such as minimizing paper and plastic usage, opting for renewable goods, and consciously lowering energy use to contribute to environmental conservation [60]. This central role of PBC in TPB highlights its significant influence on behavior, underscoring that employees with a greater sense of control are more likely to engage in pro-environmental behaviors with confidence [61]. Therefore, we propose the hypothesis as the following:

*H5: Perceived behavior control has a significant impact on employee green behavior*

### Green knowledge and employee green behavior

Green knowledge is essential for employees to engage in pro-environmental behaviors effectively, as a lack of understanding regarding environmental issues and practices can result in avoidance or hesitancy in participating in green activities [62]. Research indicates that employees who possess Green knowledge are more likely to demonstrate green creativity and engage in eco-friendly actions [51,63]. According to Levy and Marans (2012), green knowledge encompasses both knowledge of environmental issues and awareness of appropriate actions to mitigate environmental harm [64]. This implies that employees with a deeper understanding of environmental challenges and solutions are more inclined to act in ways that support sustainability, avoiding practices that may be detrimental to the environment. Studies also show that green knowledge and green behavior are mutually reinforcing, especially among individuals who are consistently aware of and involved in environmental issues [65]. Responsible environmental behavior often mirrors the extent of an individual’s green knowledge, suggesting a positive relationship between these variables [66]. Although some research suggests limited or weak interactions between green knowledge and green behavior, particularly among students in higher education [55,67], other studies underscore a stronger link in organizational contexts. For instance, green knowledge has been shown to enhance employee green behavior in various institutional settings [68], including universities in the Netherlands, where environmental awareness significantly affects sustainable behaviors [Blok et al., 2015]. Therefore, we propose the hypothesis as the following:

*H6: Green knowledge has a significant impact on employee green behavior*

### Environmental concern and employee green behavior

Environmental concern reflects the extent to which individuals recognize environmental issues and support actions to address these challenges, often through personal contributions toward solutions [69]. Employees who are environmentally concerned tend to be proactive in reducing their negative impact on the environment, such as through energy conservation efforts like shutting down electronics when not needed [70]. This sense of responsibility can drive employees to adopt behaviors that align with sustainability goals as they strive to mitigate their environmental footprint. Research supports that employees who exhibit environmental concern are more likely to engage in eco-conscious behaviors [51]. For example, studies on hotel employees in Turkey have shown that a strong environmental concern leads to heightened workplace green behaviors [6,11]. Additionally, [62,69] affirm that one’s concern for the environment is a significant driver of pro-environmental behavior. Yusliza et al. expand on this by describing environmental consciousness as a multi-faceted concept that encompasses environmental concern, which they found to significantly enhance pro-environmental behaviors among students at the training centers [71]. This viewpoint aligns with findings from Okumus et al. [11], which suggest that a strong environmental concern correlates positively with green behavior in various organizational contexts. Therefore, we propose the hypothesis as the following:

*H7: Environmental concern has a significant impact on employee green behavior*

### The mediating role of attitude

Attitude in this study refers to an employee’s beliefs to practice a specific behavior [72]. These beliefs may include concerns about environmental issues and the zeal to be a part of the solution to such problems [73]. Hence, when individuals believe in global warming as part of their environmental concern, it fosters an attitude toward environmental protection [72; 73]. In addition, the investigation pertaining to customer disposition towards green resorts in Taiwan revealed that attitude has a significant mediating role in the relationship between environmental concern and eco-friendly behavior [27]. Moreover, environmental concern is suggested to positively impact specific behavior through attitude [74]. The attitude toward a green environment also significantly mediates the association between environmental concern and the pro-environment behavior of employees in the hospitality industry [34]. Therefore, we propose the hypothesis as the following:

*H8: Attitude has a significant mediating role in the relationship between environmental concern and employee green behavior*

### The mediating role of perceived behavior

It is suggested that environmentally concerned employees with high levels of behavior control tend to practice more green behavior than employees with lower levels [59,75]. Therefore, it is posited that increased green behavior is affected by a higher perceived behavior control. Furthermore, a substantial mediating role of fear of victimization was identified in the relationship between concerns about global warming and the eco-friendly behavior of employees in the manufacturing sector in China [76]. The fear of victimization is conceptualized as the perceived behavior control in which individuals perceive fear as a motivation to implement a behavior [77]. Therefore, we propose the hypothesis as the following:

*H9: Perceived behavior control has a significant mediating role in the relationship between environmental concern and employee green behavior*

### The mediating role of green knowledge

Green knowledge is knowledge about how to use renewable energy and dispose of waste properly to reduce climate change’s impact on daily life [64]. It is claimed that green knowledge and green behavior mutually reinforce one another [65]. Hence, enhancing environmental knowledge would lead to increased environmental conduct [66]. When individuals express concerns regarding environmental pollution, they find knowledge that helps them contribute to the green solution [11,78]. Therefore, environmental knowledge serves as a mediator in the relationship between environmental concern and employee green behavior [11,44]. We propose the hypothesis as the following:

*H10: Green knowledge has a significant mediating role in the relationship between environmental concern and employee green behavior*

### Moderating role of environmental concern

Environmental concern is characterized as the extent to which individuals are aware of ecological issues, endorse initiatives to address them, and demonstrate a willingness to contribute towards their resolution [79]. Extant literature suggests that environmental concern is related to an individual’s belief that is closely associated with his/her specific attitude toward environmental issues [34]. It is also posited that an employee’s positive attitude toward ecological sustainability impacts his or her green behavior performance when his or her awareness about environmental issues is improved [3]. Therefore, employee’s attitudes toward green behavior can be developed when environmental concerns are increased. We propose the hypothesis as the following:

*H11: Environmental concerns significantly moderate the effect of attitude on employee green behavior.*

Perceived behavior control refers to individuals’ confidence in their ability to exercise control over executing a behavior within a specific context [80]. Perceived behavior control was defined as the belief individuals hold in their ability to successfully carry out a particular behavior [9]. Employees believe they can perform green behavior well if their environmental concerns are heightened [6]. Therefore, an individual’s environmental awareness significantly moderates the relationship between perceived behavior control and green behavior [81]. In addition, the outcome of some research also found the important moderating role of environmental concerns among employees in the education sector of Saudi Arabia [82]. We propose the hypothesis as the following:

*H12: Environmental concerns significantly moderate the effect of perceived behavior control on employee green behavior.*

Green knowledge is knowledge about how to use renewable energy and dispose of waste properly to reduce the impact of climate change on daily life [83]. Individuals with good green knowledge would feel confident in protecting the environment [6]. Employees who prioritize environmental concerns and aim to safeguard the environment through green behavior could gain green knowledge [62]. In other words, when employees express apprehension about the detrimental state of the ecosystem and possess a firm resolve to address it, they will actively pursue acquiring knowledge related to environmentally sustainable practices [84]. Therefore, environmental concerns moderate the association between the knowledge of climate change and green behavior [76]. We propose the hypothesis as the following:

*H13: Environmental concerns significantly moderate the effect of green knowledge on employee green behavior*

### Conceptual model

**Fig 1:**
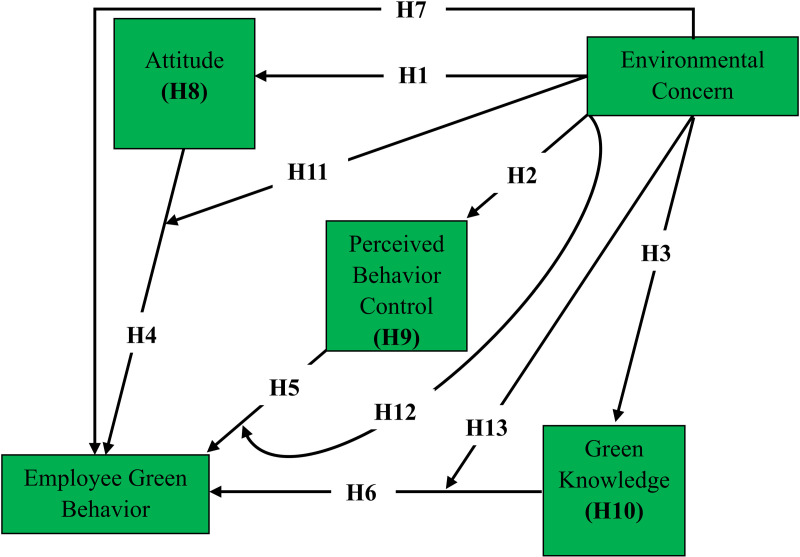
Conceptual model.

With the hypotheses developed above, we design a conceptual model as the following (Fig 1):

## Materials and methods

### Sample size determination

The Vietnam Stock Exchange (VSE), which includes all publicly listed companies in Vietnam, serves as an appropriate context for this study. The target population comprises all publicly listed Vietnamese firms, totaling 757 companies, as reported on the State Securities Commission of Vietnam’s website (www.ssc.gov.vn). No sampling method was applied in this study; thus, the entire target population was considered as the sample.

### Measures

The questionnaire employed in this study was crafted using validated measurement scales. Given that the survey was conducted in Vietnam, two bilingual academic experts fluent in both Vietnamese and English were engaged in the translation process. The questionnaire underwent pretesting in meetings with ten academic experts and ten senior managers to assess content validity and ensure that respondents clearly understood the instructions, items, and scales. A 5-point Likert scale was used throughout the questionnaire, ranging from 1 (very strongly disagree) to 5 (very strongly agree). This study received written ethical approval from the Research Ethics Committee of Van Lang University (VLREC).

The measurement of Attitude and Employee Green Behavior was adapted from the validated scale by Blok et al. [17]. Notably, Employee Green Behavior was measured using 8 items instead of the 20 items in Blok et al. [17]. This decision was made to select items that best represent this construct within the context of Vietnamese publicly listed companies while ensuring the inclusion of diverse aspects of the construct. Specifically, the authors also measured 7 aspects of Employee Green Behavior, consistent with Blok et al. [17] (Heating, Printing, Drinking, Sustainable Shopping, Computer Use, Light Use, and Recycling). However, the authors adjusted and shortened the items. For instance, most Vietnamese companies do not use heating systems due to the hot climate. Instead, air conditioners are widely used. Therefore, questions related to heating systems were replaced with questions about air conditioner usage. The Perceived Behavior Control scale was adapted from Swaim et al. [85]. The Environmental Concern scale was based on the work of Urban and Scasny [70], and the Green Knowledge scale was adapted from Gatersleben et al. [86]. All items used in the study are detailed in S1 Appendix.

### Data collection and description

A survey of publicly listed companies was conducted between April 2021 and December 2021. The targeted respondents are directors and senior managers. Four hundred fifty-five responses were directly collected from 757 distributed questionnaires. After excluding missing data and outliers based on boxplot analyses, 407 responses were retained for analysis.Table 1 below illustrates the composition of the sample based on industry type, employee count, and revenue for 2021. A significant portion of the respondents are employed in the manufacturing sector (64%). Demographic data also reveal that 74% of the companies have over 600 employees, and 83% have revenues ranging from 101 to 400 million USD. Thus, we can conclude that most of the surveyed companies are small and medium-sized enterprises.

**Table 1:** Demographic profile.

Category	Respondents (N = 407)	Non-Respondents (N = 350)	Total Population (N = 757)	Chi-Square Value	P-Value
**Type of Industry**					
Real Estate	12% (49)	15% (53)	13.5% (102)	1.22	0.27
ICT	2% (8)	4% (14)	2.9% (22)	1.97	0.16
Financial & Banking	10% (41)	9% (31)	9.5% (72)	0.14	0.71
Services	5% (20)	6% (21)	5.4% (41)	0.23	0.63
Manufacturing	64% (261)	60% (210)	62.3% (471)	1.58	0.21
Agriculture	7% (28)	6% (21)	6.5% (49)	0.27	0.6
**Number of Employees**					
<100	2% (8)	3% (11)	2.5% (19)	0.53	0.47
100-200	3% (12)	4% (14)	3.4% (26)	0.22	0.64
201-400	12% (49)	15% (52)	13.4% (101)	1.08	0.3
401-600	9% (36)	10% (35)	9.4% (71)	0.09	0.76
>600	74% (302)	68% (238)	71.3% (540)	3.17	0.08
**Revenue (million USD)**					
<50	1% (4)	2% (7)	1.5% (11)	0.91	0.34
50-100	5% (20)	6% (21)	5.4% (41)	0.23	0.63
101-200	49% (199)	45% (158)	47.1% (357)	1.16	0.28
200-400	34% (139)	36% (126)	34.9% (265)	0.38	0.54
>400	11% (45)	11% (38)	11% (83)	0	1

Source: Authors’ calculations

To ensure the robustness of the study, an analysis was conducted to compare respondents and non-respondents to address potential concerns regarding Non-Response Bias. As shown in Table 1, the demographic profiles of respondents (N = 407) closely align with those of non-respondents (N = 350) and the total surveyed population (N = 757). Statistical tests, including chi-square tests for categorical variables, confirmed no significant differences (P-values >  0.05) between respondents and non-respondents across key characteristics such as industry type, employee count, and revenue. These results indicate that the retained sample is representative of the total population and free from Non-Response Bias, ensuring the validity and generalizability of the study’s findings.

### Methods

This study employed linear moderated mediation analysis as the primary method to test the research model and hypotheses. This approach was selected for several reasons. First, the linear regression model provides a straightforward yet effective approach to assess the relationships among mediating, moderating, and dependent variables. In this study, the hypotheses were built upon the Theory of Planned Behavior (TPB), which posits linear relationships among key constructs such as attitudes, perceived behavioral control, and behavioral intentions. Specifically, environmental concerns were hypothesized to influence employees’ green behavior directly and indirectly through mediators such as attitudes, perceived behavioral control, and green knowledge. Simultaneously, the role of environmental consciousness as a moderator was examined to capture its impact on the strength of these relationships. Given the theoretical foundation, linear analysis provides a logical and suitable framework for analyzing the direct and indirect effects among variables. Moderated mediation analysis allows for a comprehensive understanding of complex relationships by evaluating both mediating and moderating effects. Additionally, linear regression is appropriate given the sample size (407 observations), ensuring linear estimates’ stability and reliability [87, 88]. This linear approach also facilitates the interpretation of complex relationships among variables, enhancing understanding of how factors like attitude, perceived behavioral control, and green knowledge influence employees’ green behavior.

The analysis was conducted using structural equation modeling (SEM) with the assistance of SmartPLS 4 software. SEM was chosen due to its ability to handle complex models involving latent constructs and multiple relationships simultaneously. The bootstrapping technique was applied to assess the significance of the direct, indirect, and moderating effects. This technique is widely recognized for its robustness in estimating standard errors and confidence intervals, ensuring reliable hypothesis testing. Collinearity among the predictor variables was assessed through Variance inflation factors (VIF) to ensure the reliability of the findings.

## Results

### The result of the construct reliability evaluation

Initially, SmartPLS 4 was utilized to assess Cronbach’s alpha (α) for reliability analysis, aimed at measuring the internal consistency of the scales used. An acceptable α value is considered to be above 0.6 [89]. Following this, exploratory factor analysis (EFA) was employed to analyze the dimensionality of the constructs and simplify the original set of items into a smaller number of composite dimensions or factors. Prior to conducting EFA, the data’s suitability was evaluated using the Kaiser-Meyer-Olkin (KMO) Test, which yielded a value of 0.905, indicating excellent sampling adequacy. Additionally, Bartlett’s Test of Sphericity was significant (p =  0.000), confirming that the correlation matrix was appropriate for factor analysis. Factor loading scores were then examined, with values meeting or exceeding the recommended threshold of 0.5 confirming the uni-dimensionality of the constructs [89]. In this study, of the original 30 measurement items, only 22 met the factor loading score threshold of 0.5, with the minimum score being 0.758. The α values for Employee Green Behavior, Attitude, Environmental Concern, Perceived Behavior Control, and Green Knowledge all exceed 0.6, ensuring the reliability of the measures. Therefore, the results in Table 2 meet the required reliability standards.

**Table 2:** Factor loading and construct reliabilities.

Factors	Factor loadings	(α)
**Employee Green Behavior**		0.874
I save energy by turning off the air-conditioning when not in the workplace	0.774	
I print and photocopy double-sided	0.880	
I recycle plastic bottle	0.831	
I turn off the computer when I leave the office	0.832	
I turn off lights when I leave the office	0.758	
**Attitude**		0.774
I support eco-friendly behavior in the workplace	0.817	
The eco-friendly behavior is important for me	0.836	
I think that the employer should support eco-friendly behavior in the workplace	0.834	
**Environmental concern**		0.934
I worry about global warming	0.877	
I worry about natural resource depletion	0.882	
I worry about water pollution	0.853	
I worry about wastewater	0.912	
I am concerned about storms and floods	0.926	
**Perceived Behavior Control**		0.919
I may control the performance of eco-friendly activities in the workplace	0.831	
I support environmental practice in the workplace	0.901	
I can perform pro-environmentally activities in the workplace	0.888	
It is convenient for me to perform eco-friendly practice	0.900	
I have my own decision about whether I perform eco-friendly activities or not	0.826	
**Green Knowledge**		0.931
I am knowledgeable about environmental issues caused by human activities	0.908	
I may see that the environment is deteriorating	0.902	
I have good knowledge about the environmental issues caused by employees in the workplace	0.922	
I am conscious of how to protect the environment from air pollution	0.906	

Source: Authors’ calculations

### The result of convergent and discriminant validity evaluation

We use SMARTPLS 4.0 to evaluate how well the model fits the data. To ensure a good fit, the overall model fitness criteria include a Standardized Root Mean Square Residual (SRMR) of 0.08 or lower, while the Normed Fit Index (NFI) should meet or exceed the threshold of 0.9 [88**].** The analysis demonstrated an acceptable model fit, with NFI at 0.907, and SRMR at 0.045.

We further employed the CFA technique to assess convergent and discriminant validity, which are essential components of construct validity. Convergent validity occurs when measurement items are highly correlated with their corresponding theoretical constructs [89]. It was evaluated using average variance extracted (AVE) and composite reliability (CRs). Convergent validity is considered satisfactory when AVE exceeds 0.5 and CRs exceeds 0.7 [89]. As shown in Table 3, the AVE values for all constructs are above 0.5, and the CRs values are above 0.7, confirming that convergent validity is met.

**Table 3:** AVE and CRs.

	CRs	AVE
Employee Green Behavior	0.909	0.666
Attitude	0.868	0.688
Environmental Concern	0.950	0.793
Perceived Behavior Control	0.939	0.756
Green Knowledge	0.950	0.827

Source: Authors’ calculations

Discriminant validity refers to the extent to which a construct is truly distinct from other constructs in the model, as indicated by low correlations between constructs. To assess discriminant validity, the Heterotrait-Monotrait (HTMT) ratio was employed, as recommended by Henseler et al. [90]. Table 4 presents the HTMT ratios for all construct pairs. The results indicate that all HTMT values are below the conservative threshold of 0.85, thereby confirming discriminant validity across constructs. This demonstrates that the constructs in the model are distinct and measure unique dimensions of the phenomenon under investigation. Consequently, the reflective construct measurements exhibit satisfactory reliability and validity.

**Table 4:** Discriminant Validity.

	EGB	ATT	EC	PBC	GK
**EGB**					
**ATT**	0.392				
**EC**	0.519	0.257			
**PBC**	0.500	0.318	0.388		
**GK**	0.287	0.412	0.209	0.256	

*Highlighted values in diagonal are square root of AVE and correlation are off-diagonal.

*Abbreviation: Employee Green Behavior: EGB; Attitude: ATT; Environmental Concern: EC;Perceived Behavior Control: PBC; Green Knowledge: GK.*

Source: Authors’ calculations.

To address concerns regarding common method bias and ensure the reliability of the study findings, a full collinearity assessment was conducted following the procedure outlined by Kock [91]. This involved adding a random single variable to the dataset and regressing it on all constructs in the model to calculate the variance inflation factor (VIF) values. Table 5 presents the VIF values for all constructs, with all values falling below the recommended threshold of 3.3, indicating that common method bias is unlikely to be a significant issue. Additionally, procedural remedies were employed during the data collection process, including ensuring respondent anonymity and using well-established measurement scales. These combined measures provide confidence that the study findings are not substantially affected by common method bias.

**Table 5:** Collinearity statistics (VIF) in Inner model.

	VIF
**ATT**	1.002
**EC**	1.325
**EGB**	1.465
**GK**	1.087
**PBC**	1.320

Source: Authors’ calculations

### The result of hypothesis tests in conceptual models

#### The test of direct and indirect effects.

The study uses the structural equation modeling (SEM) technique to test direct and indirect relationships, and the outcomes of the tests are shown in Tables 6 and 7. Firstly, as shown in Table 6, environmental concern has a positive impact on attitude (β =  0.224; p-value < 0.05), on perceived behavior control (β =  0.361; p-value < 0.05), and on green knowledge (β = 0.201; p-value < 0.05), respectively, so H1, H2, and H3 are supported. Among the three positive impacts of environmental concern on attitude, perceived behavior control, and green knowledge mentioned above, environmental concern has the most significant impact on perceived behavior control. Secondly, attitude (β =  0.149; p-value < 0.05), perceived behavior control (β =  0.298; p-value < 0.05), and environmental concern (β =  0.318; p-value < 0.05) have significant associations with employee green behavior, respectively, so, H4, H5, and H7 are supported, respectively. However, green knowledge (p-value > 0.05) does not have a significant influence on employee green behavior, so H6 is not supported.

**Table 6:** Direct effects.

Hypothesis	Β (Beta)	SE	T-value	P-values	Significance (p < 0.05)?
H1: EC => ATT	0.224	0.043	5.234	0.000	Yes
H2: EC => PBC	0.361	0.058	6.225	0.000	Yes
H3: EC => GK	0.201	0.052	3.899	0.000	Yes
H4: ATT =>EGB	0.149	0.049	3.061	0.002	Yes
H5: PBC=> EGB	0.298	0.043	6.909	0.000	Yes
H6: GK=> EGB	0.073	0.068	1.067	0.286	No
H7: EC=>EGB	0.318	0.052	6.152	0.000	Yes

*Abbreviation: Employee Green Behavior: EGB; Attitude: ATT; Environmental Concern: EC; Perceived Behavior Control: PBC; Green Knowledge: GK.*

Source: Authors’ calculations.

**Table 7:** Mediating effects.

	P-values	SE	T statistics (|O/STDEV|)	Confident interval	Significance (p < 0.05)?
H8: EC =>ATT=> EGB	0.007	0.012	2.707	[0.009,0.059]	Yes
H9: EC=> PBC=>EGB	0.000	0.025	4.385	[0.064,0.156]	Yes

*Abbreviation: Employee Green Behavior: EGB; Attitude: ATT; Environmental Concern: EC; Perceived Behavior Control: PBC.*

Source: Authors’ calculations.

Lastly, Table 7 below shows that environmental concern has indirect effects on employee green behavior through the mediating role of attitude and perceived behavior control, respectively, so H8 and H9 are supported. However, green knowledge does not have a significant effect on employee green behavior, so H6 is not supported. Thus, environmental concern also does not have an indirect effect on employee green behavior through green knowledge, so H10 is not supported.

#### The moderating effect of environmental concern.

H11, H12, and H13 proposed that the impacts of attitude, perceived behavior control, and green knowledge on employee green behavior would be positive in conditions of a high degree of the moderating role of environmental concern. However, green knowledge does not have a significant effect on employee green behavior (H6 is not supported); thus, environmental concern does not have a moderating effect on the relationship between green knowledge and employee green behavior (H13 is not supported). Therefore, we only examine H11 and H12. To test H11 and H12, the interactions of (environmental concern x attitude) and (environmental concern x perceived behavior control) are included in regression analysis. The study uses Process software to test such moderating regression analysis.

Table 8 above shows that environmental concern only moderates the relationship between perceived behavior control and employee green behavior, so H12 is supported, and H11 is not supported.

**Table 8:** Moderating effects.

	Β (Beta)	P-values	SE	T statistics (|O/STDEV|)	Confident interval	Significance (p < 0.05)?
H11: Interaction-1 => EGB	-0.079	0.115	0.050	1.579	[-0.169,0.025]	Not supported
H12: Interaction-2 => EGB	0.074	0.047	0.037	1.994	[0.000,0.147]	Yes
	**Interaction-1:** Attitude x Environmental Concern					
	**Interaction-2:** Perceived Behavior Control x Environmental Concern					

*Abbreviation: Employee Green Behavior: EGB.*

Source: Authors’ calculations.

**Fig 2:**
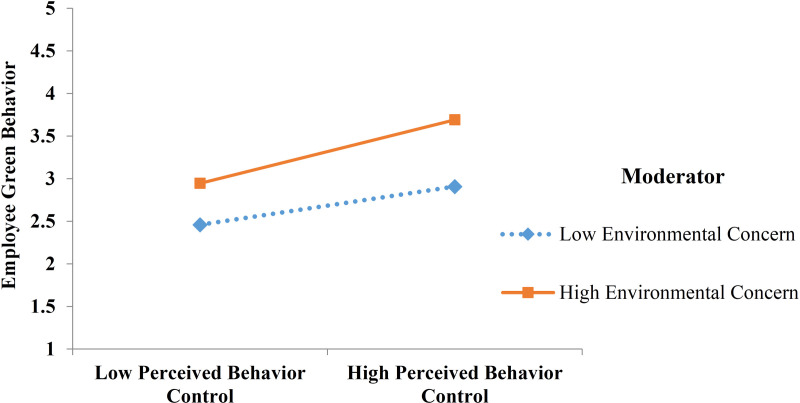
Moderating effects.

In addition, the moderating plot for significant moderators is shown in Fig 2.

## Conclusion

### Discussion

Firstly, the findings reveal that environmental concern significantly enhances employees’ attitudes towards environmental conservation, with a substantial effect size. This result aligns with previous studies highlighting the importance of environmental concern in fostering positive attitudes [32–34]. The findings suggest that employees who express a high level of concern about environmental issues are more inclined to develop a proactive mindset toward conservation efforts. This relationship reflects how deeply held concerns about environmental sustainability can shape individual perceptions and motivations, driving a more substantial commitment to pro-environmental attitudes. The relatively significant influence observed emphasizes the importance of fostering environmental awareness in corporate settings to instill positive attitudes that can serve as precursors to sustainable behavior.

Next, the paper indicates that environmental concern positively influences perceived behavioral control, aligning with previous research [33,35]. A deeper interpretation of this result suggests that individuals with heightened environmental concerns may internalize environmental values more profoundly, which bolsters their belief in their capacity to overcome barriers and implement eco-friendly practices. This aligns with the conceptual premise that perceived behavioral control reflects not only one’s confidence in performing specific behaviors but also one’s perceived ability to control the outcomes of such actions. Moreover, this relationship highlights the potential for businesses to enhance green behavior by nurturing employees’ environmental concerns. Interventions such as environmental awareness campaigns or sustainability training programs could strengthen employees’ belief in their ability to contribute effectively to environmental initiatives. These findings also support the integration of psychological constructs like perceived control into broader frameworks for understanding environmental behavior, providing valuable insights for corporate strategies aimed at promoting sustainability.

Thirdly, the study highlights a positive relationship between environmental concern and green knowledge, consistent with findings by Okumus et al. [11]. This suggests that employees who exhibit a high level of environmental concern and express distress about environmental degradation are more likely to acquire sufficient green knowledge related to environmental protection. This relationship may stem from the inherent motivation of environmentally concerned individuals to seek information and resources that enhance their understanding of sustainability practices. A closer examination of this linkage reveals that environmental concern serves as a cognitive catalyst, driving individuals to explore and internalize knowledge that aligns with their environmental values. Furthermore, this finding underscores the importance of fostering environmental concern as a precursor to knowledge acquisition in organizational settings. Companies could leverage this insight by implementing educational programs, workshops, or interactive campaigns that link environmental values to actionable knowledge. By nurturing environmental concerns, organizations can create a workforce equipped with the expertise necessary to contribute effectively to sustainability goals. Future research could delve deeper into the mechanisms that translate environmental concern into knowledge acquisition, exploring variables such as individual learning preferences and organizational support systems.

Fourthly, the empirical findings confirm a positive relationship between employees’ attitudes toward environmental protection and their engagement in green behavior, consistent with prior studies by Sabbir and Taufique [4], Fawehinmi et al. [44], and Tariq et al. [49]. Attitudes, as core components of behavioral intention within the Theory of Planned Behavior (TPB) framework, play a crucial role in driving pro-environmental actions [51]. This positive association suggests that when employees value environmental conservation and perceive it as meaningful, they are intrinsically motivated to align their actions with their beliefs. Attitudes toward environmental protection act as psychological enablers that shape how individuals interpret, prioritize, and engage in sustainability initiatives. Additionally, this finding underscores the potential for businesses to cultivate stronger environmental attitudes among their employees. Strategies such as promoting awareness campaigns, highlighting the tangible benefits of green behaviors, and recognizing environmentally responsible actions can foster positive attitudes. This, in turn, enhances employee commitment to sustainability goals.

The findings indicate that perceived behavioral control (PBC) is a strong predictor of employees’ green behavior, consistent with prior research by Arli et al. [92] and Fawehinmi et al. [3]. This suggests that employees who feel confident in their abilities and perceive themselves as having the necessary resources and opportunities to perform environmentally friendly behaviors are more likely to engage in such actions. PBC, as a key component of the Theory of Planned Behavior (TPB), reflects an individual’s perception of the ease or difficulty of performing a particular behavior. When employees have higher levels of PBC, they are better equipped to overcome potential obstacles and are more flexible in integrating green practices into their daily work routines. For example, confidence in managing waste disposal effectively or adopting energy-saving techniques at the workplace can significantly motivate employees to practice sustainable behaviors. This finding underscores the importance of organizational interventions in enhancing PBC. Employers can provide training programs to build environmental skills, ensure the availability of necessary tools and resources for green practices, and create a supportive work environment that reinforces employee autonomy and competence in sustainability-related tasks. Such initiatives can enhance employees’ belief in their capability to engage in green behaviors, thereby fostering a culture of environmental responsibility.

The findings reveal no significant indirect relationship between environmental concern and employees’ green behavior mediated through green knowledge. This result diverges from prior studies by Fawehinmi et al. [44] and Safari et al. [93], which suggest that green knowledge is a critical intermediary in fostering green behavior. The absence of this linkage may indicate that possessing environmental knowledge alone does not translate directly into actionable green practices among employees. A plausible explanation for this discrepancy is that employees might possess only superficial or general knowledge about environmental issues without adequate training or guidance on how to implement specific eco-friendly behaviors effectively. For example, understanding the importance of energy conservation may not inherently equip employees with the skills to optimize energy use at work. The lack of actionable knowledge or practical training could diminish the transformative potential of green knowledge on green behavior. This finding underscores the importance of developing structured and actionable training programs that go beyond general environmental awareness. Businesses should design targeted interventions, such as workshops, practical demonstrations, and detailed guidelines, to empower employees with specific skills and strategies for environmental protection. Additionally, fostering a supportive corporate culture that prioritizes sustainability can help bridge the gap between environmental knowledge and actual green behavior.

Seventhly, the findings demonstrate that environmental concern positively influences employees’ green behavior, aligning with prior studies such as Ahmed et al. [6], Fawehinmi et al. [44], Farooq et al. [51], and Okumus et al. [11]. These studies collectively suggest that employees with heightened awareness and concern for environmental issues are more likely to engage in eco-friendly actions and behaviors within their workplaces. This result highlights the pivotal role of environmental concern as a motivational driver. Employees who perceive environmental degradation as a pressing issue are more likely to internalize the importance of sustainable practices, leading to proactive engagement in green behaviors. Such behaviors may include energy conservation, waste reduction, and participation in organizational sustainability initiatives. The positive linkage between environmental concern and green behavior may also reflect the growing impact of environmental values on individual decision-making processes. Employees driven by strong ecological values are not only motivated to act sustainably but may also influence their peers and organizational culture, fostering a collective commitment to sustainability. From a practical perspective, businesses can capitalize on this relationship by cultivating and reinforcing environmental concerns among employees. This could involve campaigns to raise awareness about environmental issues, integrating sustainability into corporate values, and recognizing or rewarding green behaviors. Furthermore, embedding environmental concerns into corporate training and development programs could amplify their impact, ensuring that employees are both aware of and equipped to act on their environmental values.

Eighthly, the results highlight that attitude and perceived behavioral control (PBC) play mediating roles in the relationship between environmental concern and employees’ green behavior. This aligns with prior research, particularly Chen and Tung [27], which underscores how these psychological constructs facilitate the translation of environmental concerns into actionable behaviors. The mediation by attitude suggests that employees’ environmental concern influences the formation of a positive mindset toward engaging in eco-friendly practices. When individuals internalize environmental issues as significant, their attitudes toward green behavior become more favorable, fostering higher motivation to act sustainably. This process emphasizes the importance of shaping attitudes to strengthen the link between concern and behavior. Similarly, the mediating role of PBC indicates that employees with heightened environmental concerns perceive greater self-efficacy and control over their ability to perform green behaviors. This reflects the constructs within the Theory of Planned Behavior [22], which posits that PBC directly influences behavioral intentions by reinforcing individuals’ confidence in overcoming barriers to sustainable practices. These findings contribute to the literature by demonstrating that environmental concern may not only directly lead to green behavior but exert its influence through intermediary psychological factors. This dual mediation model broadens our understanding of how green behaviors emerge, providing a nuanced perspective that bridges individual motivation and actual behavior.

Finally, the study’s most compelling finding reveals that environmental concern significantly moderates the relationship between perceived behavioral control (PBC) and employees’ green behavior. This result supports the insights of Kautish et al. [81], who emphasize that employees with a heightened sense of confidence are more likely to engage in green behaviors, particularly when they possess a strong awareness of and concern for environmental issues. The moderating role of environmental concern suggests that it amplifies the positive effects of PBC on green behavior, acting as a catalyst for environmentally conscious actions. This finding highlights the dynamic interplay between cognitive and affective components of pro-environmental behavior. While PBC reflects an individual’s confidence and perceived capacity to act, environmental concern integrates a deeper emotional and value-driven commitment to environmental protection. Together, these factors create a synergy that reinforces sustainable behavior, as employees who care deeply about the environment are likely to align their sense of control with their personal values. From a theoretical standpoint, this study enriches the literature on pro-environmental behavior by demonstrating the conditional role of environmental concern in shaping the PBC-behavior linkage. It underscores the need to incorporate both motivational and emotional dimensions in models predicting green behavior, providing a more comprehensive understanding of the factors driving sustainable practices. Practically, these findings suggest that businesses should foster an environment that not only builds employees’ confidence in their ability to perform green actions but also heightens their awareness and concern for environmental issues. This could be achieved through targeted training, sustainability campaigns, and policies emphasizing individual contributions to broader environmental goals.

### Theoretical contribution

While the Theory of Planned Behavior (TPB) has been widely used to study behavioral intentions, its application in understanding green behavior in corporate environments—particularly in emerging economies—remains underexplored. By focusing on managerial and senior employees in publicly listed companies in Vietnam, the study provides valuable contextual insights into an essential but under-researched demographic. With its unique socio-economic and cultural characteristics, Vietnam presents a critical testbed for theories of green behavior that have been mainly developed in Western contexts. This contextualization enriches the generalizability of theoretical frameworks and addresses the call for more research within varied environments and emerging economies.

One of this study’s most novel contributions is identifying environmental concern as a significant moderator in the relationship between perceived behavioral control and green behavior. This finding suggests that employees with heightened environmental concerns are better equipped to leverage their confidence and control over work processes to engage in sustainable practices. This result advances TPB by demonstrating that environmental concern is not only a direct predictor of employee green behavior but also a crucial moderating factor in the relationship between perceived behavior control and green behavior. This dual role of environmental concern extends TPB’s applicability and opens new avenues for integrating values-driven constructs into behavioral models in environmental psychology.

This research challenges traditional assumptions within the TPB framework. While prior studies have emphasized the direct impact of perceived behavioral control, attitude, and green knowledge on employee green behavior, our findings reveal that green knowledge does not significantly predict green behavior in the studied context. This suggests that employees may struggle to translate awareness into tangible behaviors without supportive mechanisms such as training or organizational systems that enable the practical application of knowledge. This nuanced result critically reconsiders the TPB’s predictive variables, suggesting that green behavior may depend more heavily on attitudinal and control-related factors than on mere knowledge. This insight is critical for developing more effective strategies to bridge the gap between knowledge and action.

The findings of this study encourage a more integrated approach to understanding employee green behavior by combining elements of TPB with moderating and mediating perspectives. The study also paves the way for future research to investigate similar dynamics in other cultural and organizational settings, as well as explore additional factors that may condition or mediate the relationship between environmental concern and green behavior. These contributions not only advance theoretical discourse but also provide a foundation for developing practical interventions aimed at fostering environmental sustainability within corporate environments.

### Practical contribution

From an industry perspective, this study offers actionable insights for enhancing environmental sustainability in the workplace. By implementing structured green initiatives, employers are encouraged to cultivate a corporate culture centered on environmental conservation. These initiatives might include detailed employee training programs on environmentally conscious practices, such as using eco-friendly transportation, conserving energy by switching off unused devices and leveraging virtual meetings to reduce travel-related emissions. Establishing such practices can foster a workplace environment where sustainable behavior becomes the norm.

The findings also highlight the importance of balancing efforts to enhance employees’ attitudes toward environmental protection and their environmental awareness. Although attitude exhibits a less pronounced predictive power than environmental concern, both factors contribute meaningfully to green behavior. Consequently, management should consider allocating resources equitably to campaigns or programs that simultaneously bolster employee awareness and foster positive environmental attitudes.

Additionally, the study underscores the pivotal role of perceived behavior control in shaping green behavior, particularly when amplified by high levels of environmental concern. Employers can harness this insight by creating an empowering environment that enhances employees’ confidence and ability to enact green practices. This could involve streamlining processes that allow employees to engage in sustainable practices with minimal effort or providing platforms for employee-led green initiatives. Green education and training programs remain a critical avenue to enrich employees’ knowledge, attitudes, and behaviors, embedding sustainability into organizational culture.

For policymakers, the study offers valuable contributions to promoting environmentally sustainable development. Policymakers should consider mechanisms that not only increase employees’ environmental concerns but also translate these concerns into actionable outcomes by fostering positive attitudes and perceived behavior control. Organizing large-scale public campaigns to raise environmental awareness can serve as a foundation for this effort, influencing individual and organizational behavior alike.

Furthermore, policymakers can incentivize businesses to adopt sustainability-focused practices by offering financial benefits, such as subsidies or tax breaks, for implementing green training programs or integrating eco-friendly practices into daily operations. These incentives can motivate employers to invest in mechanisms that encourage employees to consistently engage in environmentally conscious behaviors, contributing to broader sustainability goals.

### Limitations and further research

The paper has limitations that should be considered when interpreting the results, and these limitations also open avenues for further research. First, the paper did not examine the concepts of green self-efficacy that may extend the current results. Therefore, further research will focus more on how green self-efficacy influences employee green behavior. Second, the article provides evidence proving that green knowledge does not significantly impact green behavior, so green knowledge is not overlooked in this study. Further study needs to investigate how green human resource management moderates the relationship between green knowledge and employee green behavior. Lastly, the research method applied in this study is the quantitative method, and future research may use qualitative methods to provoke theoretical support for employee green behavior.

## Supporting information

S1 Appendix Survey questionnaire.
(DOCX)

S1 File Dataset used in analysis.
(XLSX)
