# Supplementary material for: Unleashing the green potential: Unraveling the power of environmental concerns in driving employees’ green behavior
Source: PLoS One. 2025 Mar 25;20(3):e0320053. doi: 10.1371/journal.pone.0320053 (PMC11936254; doi:10.1371/journal.pone.0320053)
Supplement: S1 Appendix — (DOCX) [file pone.0320053.s001.docx]

**S1 Appendix. Questionnaire items**

| **Factors** | **Questionnaire items** |
| --- | --- |
| **Employee green behavior** |  |
| Employee green behavior 1 | I save energy by turning off the air-conditioning when not in the workplace |
| Employee green behavior 2 | I print and photocopy double-sided |
| Employee green behavior 3 | I recycle plastic bottle |
| Employee green behavior 4 | I turn off the computer when I leave the office |
| Employee green behavior 5 | I turn off lights when I leave the office |
| Employee green behavior 6 | I save water when washing dishes and cups |
| Employee green behavior 7 | I share knowledge about environmental protection with my colleagues |
| Employee green behavior 8 | I buy an eco-friendly car or motorcycle |
| **Attitude** |  |
| Attitude 1 | I support eco-friendly behavior in the workplace |
| Attitude 2 | The eco-friendly behavior is important for me |
| Attitude 3 | I think that the employer should support eco-friendly behavior in the workplace |
| Attitude 4 | I think that the company should provide environmental protection training courses to employees |
| Attitude 5 | I suggest the company build environmentally friendly buildings |
| **Environmental concern** |  |
| Environmental concern 1 | I worry about global warming |
| Environmental concern 2 | I worry about natural resource depletion |
| Environmental concern 3 | I worry about water pollution |
| Environmental concern 4 | I worry about wastewater |
| Environmental concern 5 | I am concerned about storms and floods |
| Environmental concern 6 | I worry about the greenhouse effect |
| **Perceived behavioral control** |  |
| Perceived behavioral control 1 | I may control the performance of eco-friendly activities in the workplace |
| Perceived behavioral control 2 | I support environmental practice in the workplace |
| Perceived behavioral control 3 | I can perform pro-environmentally activities in the workplace |
| Perceived behavioral control 4 | It is convenient for me to perform eco-friendly practice |
| Perceived behavioral control 5 | I have my own decision about whether I perform eco-friendly activities or not |
| **Green Knowledge** |  |
| Green Knowledge 1 | I am knowledgeable about environmental issues caused by human activities |
| Green Knowledge 2 | I may see that the environment is deteriorating |
| Green Knowledge 3 | I have good knowledge about the environmental issues caused by employees in the workplace |
| Green Knowledge 4 | I am conscious of how to protect the environment from air pollution |
| Green Knowledge 5 | I suggest that we should educate the next generation about green knowledge |
| Green Knowledge 6 | I suggest that we should organize this meeting about green and clean living in the company |
